# Supplementary material for: The impact of doctor–patient communication on medication adherence and blood pressure control in patients with hypertension: a systematic review
Source: PeerJ. 2024 Nov 28;12:e18527. doi: 10.7717/peerj.18527 (PMC11608562; doi:10.7717/peerj.18527)
Supplement: Supplemental Information 2 [file peerj-12-18527-s002.docx]

**Pubmed**

| # | Query | |
| --- | --- | --- |
|  | Search | (hypertension OR (Blood Pressure, High) OR (Blood Pressures, High) OR (High Blood Pressures) OR (High Blood Pressure) [All fields] |
|  | Search | (Communication) OR (Social Communication) OR (Communication, Social) OR (Communications, Social)) OR (Social Communications) OR (Misinformation) OR (Personal Communication) OR (Communication, Personal)) OR (Communication Programs) OR (Communication Program) OR (Program, Communication) OR (Programs, Communication) OR (Communications Personnel) OR (Personnel, Communications) [All fields] |
|  | Search | (Doctor) OR (physician) OR (physicians) OR (General Practitioners) [All fields] |
|  | Search | (Medication Adherence) OR (Adherence, Medication) OR (Drug Adherence) OR (Adherence, Drug) OR (Medication Nonadherence) OR (Nonadherence, Medication) OR (Medication Noncompliance) OR (Noncompliance, Medication) OR (Medication Non-Adherence) OR (Non-Adherence, Medication)) OR (Medication Persistence) OR (Persistence, Medication) OR (Medication Compliance) OR (Compliance, Medication) OR (Medication Non-Compliance)) OR (Non-Compliance, Medication) OR (Drug Compliance) OR (Compliance, Drug) [All fields] |
|  | Search | (1 and 2 and 3 and 4) |

**Web of Science [Clarivate]**

Search in: All Databases; Collections: Web of Science; MEDLINE

| # | Query |
| --- | --- |
|  | **(TS=(hypertension)) OR TS=(Blood Pressure, High) OR TS=(Blood Pressures, High) OR TS=(High Blood Pressures) OR TS=(High Blood Pressure)** |
|  | **(TS=(Communication) OR TS=(Social Communication) OR TS=(Communication, Social) OR TS=(Communications, Social) OR TS=(Social Communications) OR TS=(Misinformation) OR TS=(Personal Communication) OR TS=(Communication, Personal) OR TS=(Communication Programs) OR TS=(Communication Program) OR TS=(Program, Communication)) OR TS=(Programs, Communication) OR TS=(Communications Personnel) OR TS=(Personnel, Communications)** |
|  | **(TS=(Doctor)) OR TS=(physician) OR TS=( physicians) OR TS=(General Practitioners)** |
|  | **(TS=(Medication Adherence) OR (TS=(Adherence , Medication) OR TS=(Drug Adherence)) OR TS=(Adherence, Drug) OR TS=(Medication Nonadherence) OR TS=(Nonadherence, Medication) OR TS=(Medication Noncompliance) OR TS=(Noncompliance, Medication) OR TS=(Medication Non-Adherence) OR TS=(Non-Adherence, Medication) OR TS=(Medication Persistence) OR TS=(Persistence, Medication) OR TS=(Medication Compliance) OR TS=(Compliance, Medication) OR TS=(Medication Non-Compliance) OR TS=(Non-Compliance, Medication) OR TS=(Drug Compliance) OR TS=(Compliance, Drug)** |
|  | (1 and 2 and 3 and 4) |

**Embase**

| # | Query |
| --- | --- |
|  | (hypertension OR 'blood pressure, high' OR 'blood pressures, high' OR 'high blood pressures' OR 'high blood pressure') |
|  | (communication OR 'social communication' OR 'communication, social' OR 'communications, social' OR 'social communications' OR 'misinformation' OR 'personal communication' OR 'communication, personal' OR 'communication programs' OR 'communication program' OR 'program, communication' OR 'programs, communication' OR 'communications personnel' OR 'personnel, communications') |
|  | (doctor OR physician OR physicians OR 'general practitioners') |
|  | ('medication adherence' OR 'adherence, medication' OR 'drug adherence' OR 'adherence, drug' OR 'medication nonadherence' OR 'nonadherence, medication' OR 'medication noncompliance' OR 'noncompliance, medication' OR 'medication non-adherence' OR 'non-adherence, medication' OR 'medication persistence' OR 'persistence, medication' OR 'medication compliance' OR 'compliance, medication' OR 'medication non-compliance' OR 'non-compliance, medication' OR 'drug compliance' OR 'compliance, drug') |
|  | (1 and 2 and 3 and 4) |

**Cochrane Library**

| # | Query |
| --- | --- |
|  | (hypertension):ti,ab,kw OR (Blood Pressure, High):ti,ab,kw OR (Blood Pressures, High):ti,ab,kw OR (High Blood Pressures):ti,ab,kw OR (High Blood Pressure):ti,ab,kw |
|  | (Communication):ti,ab,kw OR (Social Communication):ti,ab,kw OR (Communication, Social):ti,ab,kw OR (Communications, Social):ti,ab,kw OR (Social Communications):ti,ab,kw OR (Misinformation):ti,ab,kw OR (Personal Communication):ti,ab,kw OR (Communication, Personal):ti,ab,kw OR (Communication Programs):ti,ab,kw OR (Communication Program):ti,ab,kw OR (Program, Communication):ti,ab,kw OR (Programs, Communication):ti,ab,kw OR (Communications Personnel):ti,ab,kw OR (Personnel, Communications):ti,ab,kw |
|  | (Adherence, Medication):ti,ab,kw OR (Drug Adherence):ti,ab,kw OR (Medication Adherence):ti,ab,kw OR (Adherence, Drug):ti,ab,kw OR (Medication Nonadherence):ti,ab,kw OR (Nonadherence, Medication):ti,ab,kw OR (Medication Noncompliance):ti,ab,kw OR (Medication Noncompliance):ti,ab,kw OR (Medication Non-Adherence):ti,ab,kw OR (Non-Adherence, Medication):ti,ab,kw OR (Medication Persistence):ti,ab,kw OR (Persistence, Medication):ti,ab,kw OR (Medication Compliance):ti,ab,kw OR (Medication Compliance):ti,ab,kw OR (Medication Non-Compliance):ti,ab,kw OR (Non-Compliance, Medication):ti,ab,kw OR (Drug Compliance):ti,ab,kw OR (Compliance, Drug):ti,ab,kw |
|  | (Doctor):ti,ab,kw OR (physician):ti,ab,kw OR (physicians):ti,ab,kw OR (General Practitioners):ti,ab,kw |
|  | (1 and 2 and 3 and 4) |

**EBSCO**

| # | Query |
| --- | --- |
| 1 | hypertension OR Blood Pressure, High OR Blood Pressures, High OR High Blood Pressures OR High Blood Pressure |
| 2 | Communication OR Social Communication OR Communication, Social OR Communications, Social OR Social Communications OR Misinformation OR Personal Communication OR Communication, Personal OR Communication Programs OR Communication Program OR Program, Communication OR Programs, Communication OR Communications Personnel OR Personnel, Communications |
| 3 | Medication Adherence OR Adherence, Medication OR Drug Adherence OR Adherence, Drug OR Medication Nonadherence OR Nonadherence, Medication OR Medication Noncompliance OR Noncompliance, Medication OR Medication Non-Adherence OR Non-Adherence, Medication OR Medication Persistence OR Persistence, Medication OR Medication Compliance OR compliance medication OR Medication Non-Compliance OR Non-Compliance, Medication OR drug compliance OR Compliance, Drug |
| 4 | Doctor OR physician OR physicians OR General Practitioners |
| 5 | (1 and 2 and 3 and 4) |
